# Serum Markers of Inflammation Mediate the Positive Association Between Neuroticism and Depression

**DOI:** 10.3389/fpsyt.2018.00609

**Published:** 2018-11-20

**Authors:** Frank M. Schmidt, Christian Sander, Juliane Minkwitz, Roland Mergl, Bethan Dalton, Lesca M. Holdt, Daniel Teupser, Ulrich Hegerl, Hubertus Himmerich

**Affiliations:** ^1^Department of Psychiatry and Psychotherapy, University Hospital Leipzig, Leipzig, Germany; ^2^Leipzig University Medical Center, IFB Adiposity Diseases, Leipzig, Germany; ^3^Department of Psychological Medicine, King's College London, London, United Kingdom; ^4^Institute of Laboratory Medicine, University Hospital Munich, LMU Munich, Munich, Germany

**Keywords:** neuroticism, depression, chronic stress, cytokines, TNF-α, mediation analyses

## Abstract

**Background:** The personality trait neuroticism has been implicated in a poor response to stress, may relate to increased concentrations of cytokines and the development of depression. Inflammatory mechanisms may also be associated with the onset, severity and symptoms of depression. Both are related to poor antidepressant treatment outcome. Therefore, mediators of inflammation may bridge the relationship between neuroticism and depression.

**Methods:** To disentangle these interrelationships, the associations between neuroticism (according to NEO-PIR-N), depressive symptoms (BDI-II scores) and serum levels of hsCRP, TNF-α, IFN-γ, IL-2, IL-4, IL-5, IL-10, IL-12, IL-13, GM-CSF were investigated in a group of 212 participants, consisting of 37 depressed and 175 non-depressed subjects. A mediation model was used to investigate whether the impact of neuroticism on depressive symptoms may be mediated by cytokines.

**Results:** Regression analyses revealed that IFN-γ, IL-5, and IL-12-levels, but none of the anti-inflammatory cytokines, were associated with the overall neuroticism score and several of the cytokines were related to the different facets of neuroticism. TNF-α, IFN-γ, IL-5, IL-12, and IL-13 were further related to the severity of depressive symptoms, as well as the somatic-affective and the cognitive dimensions of depression. Pro-inflammatory IFN-γ, IL-5 and IL-12 were identified as mediators of the positive prediction of depression severity by the degree of neuroticism.

**Conclusions:** The current findings demonstrate that conditions related to long-term stress, such as depression and high neuroticism, are related to an up-regulation of inflammatory agents. Neuroticism may increase stress perception and, thus, increase the production of pro-inflammatory messenger molecules which are involved in the development of depression. This evidence may contribute to future anti-inflammatory interventions, particularly in subjects with high neuroticism who are at risk for developing depression. Furthermore, depressed patients with high neuroticism and cytokine levels may require early escalations in the intensity of treatment, along with additional therapeutic elements to increase the rate of treatment success.

## Introduction

Neuroticism, as one of the Big Five higher-order personality traits, represents the tendency to experience negative emotions, such as anxiety and anger, and to have an increased perception of stress, as well as the inability to relieve the self from and to cope with stress (1). Acute and chronic stress have been shown to significantly influence cytokine production in human and animal studies (2, 3). In accord, higher neuroticism as well as stress-related disorders, such as post-traumatic stress disorder (PTSD), have been found to be associated with higher levels of pro-inflammatory agents (4, 5). However, investigations on neuroticism have been limited to a few inflammatory parameters: for example, C-reactive protein (CRP) or interleukin (IL)-6, for which the relationship with neuroticism could not be confirmed (6). Importantly, neuroticism is considered a risk factor for the development and onset of major depression and certain subtypes of depression (7–9). However, the biological mechanisms for this relationship are not yet well understood.

Alongside neuroticism, a body of evidence supports the involvement of low-grade inflammation in the pathogenesis of depressive disorders (10). Accordingly, cross-sectional studies have demonstrated an association between pro-inflammatory markers and the presence, course and treatment outcome of major depression (11–14). Inconsistent results have been observed in the relationship between pro-inflammatory cytokines and the severity of depressive symptoms, as measured in cohorts of patients with major depression or physical illnesses concomitant with depressive syndromes, and in population-based studies (15–22). Additionally, anti-inflammatory cytokines have gained little attention in these investigations (15, 23). The relationship between the symptoms and severity of depression and cytokine regulation should be interpreted with caution given that the majority of investigations were limited to a few parameters, in particular CRP and IL-6, and relevant confounders, such as the distribution of sexes, have not always been taken into account sufficiently.

Given that only a small number of mediators of inflammation have yet been investigated with regards to neuroticism, results on inflammation and depressive symptomatology are inconclusive and the biological pathways for the relationship between neuroticism and depression are unidentified. Therefore, the aim of this study was firstly to investigate the hypothesis that pro- and anti-inflammatory markers are associated with the severity of depressive symptoms and the personality trait neuroticism. Secondly, we aimed to explore if cytokines mediate the relationship between depressive symptoms and neuroticism.

## Methods

### Participants

The presented data were collected as part of the “OBDEP” research project (Obesity and Depression: pathogenetic role of sleep and wakefulness regulation, motor activity level and neurochemical aspects). Three-hundred and four participants were initially recruited from the outpatient clinic of the Integrated Research and Treatment Center (IFB) for Adiposity Diseases Leipzig, from the Department of Psychiatry and Psychotherapy at the University Hospital Leipzig and via advertisements (intranet, internet, local newspapers). Evaluation of inclusion and exclusion criteria for the study was performed in two stages, as previously reported (24). First, potential participants took part in a telephone screening interview, which involved collecting socio-demographic data, assessing the presence of physical illnesses and completing a checklist of the Structured Clinical Interview for DSM-IV [SCID-I; (25)]. Following this, potentially eligible participants were invited to the study center, where exclusion criteria were assessed in more detail. In cases of positive SCID-screening or known diagnosis of depression, the SCID-I was performed in full. Exclusion criteria were DSM-IV Axis 1 disorders for the non-depressed participants and DSM-IV Axis 1 disorders other than a major depression for the depressed subjects. For all subjects, further exclusion criteria were acute or chronic infections (according to clinical investigation and/or CRP ≥ 10 mg/l), current medication treatment with a non-steroidal anti-inflammatory drug (NSAID), the presence of current and/or past neurological disorders, and a history of head injury with loss of consciousness exceeding 1 h. Assessments for current and past history of health problems and current medication were performed using standardized questionnaires. Only data sets of participants who completed both the Beck Depression Inventory II [BDI-II; (26)] and the Revised NEO Personality Inventory [NEO-PIR-N; (27)] were included in the statistical analyses presented herewith, resulting in a total sample of 212 participants, including 175 non-depressed and 37 depressed subjects. All participants were aged 18–70 years. Written informed consent was obtained from all participants. The study was approved by Leipzig University Ethics Committee (#015-10-18012009).

### Assessments

Depressive symptoms were assessed using the Beck Depression Inventory II [BDI-II; (26)]. Along with a total sum score, sub-scale scores for a somatic-affective factor and a cognitive factor were calculated (28). The somatic-affective factor was calculated by summation of scores on items 4, 11-13, and 15-21 and the cognitive factor by summation of scores on items 1-3, 5-9, and 14. To assess neuroticism, the neuroticism domain (consisting of six dimensions: anxiety, hostility, depression, self-consciousness, stress vulnerability, impulsiveness) of the Revised NEO Personality Inventory [NEO-PIR-N; (27)] was used.

### Cytokine measurement

After blood drawing, serum probes were immediately centrifuged at 3,000 rpm for 10 min. The supernatant was aliquoted and stored in non-absorbing polypropylene tubes of 300 μl. Probes were shock-frozen in liquid nitrogen and stored in freezers at −80°C until required. Cytokines were measured using the Bio-Plex Pro human cytokine Th1/Th2 immunoassay from Bio Rad, Germany, a 96-well kit that uses coupled magnetic beads and detection antibodies. This multiplex assay detects IL-2, IL-4, IL-5, IL-10, IL-12, IL-13, GM-CSF, IFN-γ, and TNF-α. The intraassay coefficient of variation (CV) for cytokines was between 1.6 and 3.8%. High sensitivity (hs)-CRP was measured using a turbidimetric assay on an AU 5800 analyzer, Beckman Coulter, Germany. For hs-CRP intraassay CV was 4.1%.

### Statistics

CRP and the nine pro- and anti-inflammatory cytokines were log-transformed in order to obtain approximately normally distributed variables. We investigated the association between depressive symptoms and neuroticism with the log-transformed serum concentrations of CRP and the cytokines by linear regression analyses.

Multiple regression analyses were performed to determine the relationship between the dependent variable (i.e., depressive symptoms according to BDI-scores) and each of the log-transformed CRP and cytokine values. Regression analyses were controlled for confounding variables known to impact cytokine levels: age, sex, smoking status, BMI and time of blood drawing. Sum values of the physical illnesses and medication listed in Table 1 were further included as control variables ranging from 0 (“none”) to the maximum of 4. Levels of significance were adjusted for multiple testing using the False Discovery Rate [FDR; according to (29)] for BDI-II, NEO-PIR-N and the respective cytokines separately. For all other analyses, the level of significance was set at *p* < 0.05. Mediation analyses were performed for those cytokines significantly associated with sum scores of both BDI-II and NEO-PIR-N. For the mediation analyses, residuals were calculated for logTNF-α, logIFN-γ, logIL-5, logIL-12, logIL-13, BDI-II sum scores and NEO-PIR-N sum scores through listwise-regression analyses in order to exclude significant confounders. Covariates included into the analyses were: “age,” “sex,” “BMI,” “smoking status,” “time of blood sampling,” “sum of physical illnesses,” and “sum of medication.” After excluding multicollinearity between the parameters, mediation analyses were performed with the freely available SPSS macro “sobel” (http://www.processmacro.org/download.html), which includes bootstrapping and Sobel test (30). All further analyses were performed with SPSS Version 24 (IBM Corporation; Armonk, NY, USA).

**Table 1:** Sociodemographic details for the total sample, and for participants with and without a diagnosis of major depression, separately.

	**Participants total**	**Participants non-depressed**	**Participants depressed**
	**(*N* = 212)**	**(*N* = 175)**	**(*N* = 37)**
Sex [male/female]	81/131	62/113	19/18
Age [years] (mean ± SD)	37.14 ± 12.01	36.61 ± 13.04	39.62 ± 12.73
Smoker (yes/no)	48/164	37/138	11/26
BMI [kg/m2] (mean ± SD)	34.71 ± 13.00	35.28 ± 12.25	32.02 ± 10.61
**Marital status**			
married or cohabiting	53 (25.0%)	39 (22.3%)	14 (37.8%)
Divorced	23 (10.8%)	17 (9.7%)	6 (16.2%)
Widowed	3 (1.4%)	2 (1.1%)	1 (2.7%)
Single	131 (61.8%)	115 (65.7%)	16 (43.2%)
NA	1.2%	2 (1.2%)	0
**Occupational status**			
Employed	106 (50.0%)	85 (48.6%)	21 (56.8%)
Unemployed	47 (22.2%)	38 (21.7%)	9 (24.3%)
Retired	12 (5.7%)	8 (4.6%)	4 (10.8%)
Student	40 (18.9%)	37 (21.1%)	3 (8.1%)
Homemaker	4 (1.9%)	4 (2.3%)	0
NA	3 (1.4%)	3 (1.7%)	0
**Comorbid Disorders (yes)**	87 (41.0%)	73 (41,7%)	14 (37,8%)
None	125 (59.0%)	102 (58.3%)	23 (62.2%)
Arterial hypertension	50 (23.6%)	42 (24.0%)	8 (21.6%)
Hypothyreosis	36 (16.9%)	31 (17.7%)	5 (13.5%)
Diabetes	25 (11.8%)	24 (13.7%)	1 (2.7%)
Obstructive sleep apnea syndrome	8 (3.8%)	7 (4.0%)	1 (2.7%)
Asthma	7 (3.3%)	6 (4.1%)	1 (2.7%)
Cerebrovascular disorders/Myocardial infarction	12 (5.7%)	8 (4.6%)	4 (10.8%)
**Medication (yes)**	60 (28.3%)	52 (29.7%)	8 (21.6%)
None	152 (71.7%)	123 (70.3%)	29 (78.4%)
ACE-blocker	28 (13.2%)	26 (14.9%)	2 (5.4%)
Beta-blocker	25 (11.8%)	22 (12.6%)	3 (8.1%)
Hypoglycaemics	25 (11.8%)	24 (13.7%)	1 (2.7%)
AT1-blocker	18 (8.5%)	12 (6.9%)	5 (13.5%)
Calcium chanel blocker	9 (4.2%)	7 (5.7%)	2 (5.4%)
Statins	3 (1.4%)	3 (1.7%)	0

## Results

Two hundred twelve participants, consisting of 37 depressed and 175 non-depressed subjects, were included in the final analyses. Demographic data, including medication status and the presence of physical illnesses, are presented in Table 1. Serum concentrations of CRP and cytokines, depression symptoms and neuroticism scores are shown in Table 2.

**Table 2:** Sum total and dimension scores for depression and neuroticism, and concentrations of markers of inflammation in the total sample, and for participants with and without a diagnosis of major depression, separately.

	**Participants total**	**Participants non-depressed**	**Participants depressed**
	**(*N* = 212)**	**(*N* = 175)**	**(*N* = 37)**
BDI-II sum score (mean ± SD)	11.01 ± 11.34	7.65 ± 7.81	26.95 ± 11.94
Somatic-affective dimension (mean ± SD)	6.21 ± 6.37	4.33 ± 4.38	15.63 ± 6.47
Cognitive dimension (mean ± SD)	4.14 ± 4.89	2.98 ± 3.64	10.33 ± 5.98
NEO-PIR-N sum score (mean ± SD)	89.15 ± 28.01	83.23 ± 25.59	117.14 ± 21.39
Anxiety dimension (mean ± SD)	15.23 ± 6.04	14.03 ± 5.51	20.89 ± 5.17
Hostility dimension (mean ± SD)	15.15 ± 5.49	14.30 ± 5.19	19.16 ± 5.10
Depression dimension (mean ± SD)	14.49 ± 7.55	12.76 ± 6.70	22.65 ± 5.60
Self-consciousness dimension (mean ± SD)	16.56 ± 5.57	15.66 ± 5.35	20.81 ± 4.59
Impulsiveness dimension (mean ± SD)	14.72 ± 4.11	14.73 ± 4.14	14.68 ± 4.02
Stress vulnerability dimension (mean ± SD)	13.00 ± 6.05	11.75 ± 5.28	18.95 ± 5.95
CRP levels [mg/dl] (mean ± SD)	0.68 ± 0.78	0.69 ± 0.76	0.65 ± 0.85
TNF-α levels [pg/ml] (mean ± SD)	30.37 ± 18.02	28.69 ± 16.56	38.29 ± 22.34
IFN-γ levels [pg/ml] (mean ± SD)	149.67 ± 113.18	119.58 ± 67.38	292.97 ± 167.10
IL-2 levels [pg/ml] (mean ± SD)	6.46 ± 8.13	6.46 ± 8.42	6.46 ± 6.71
IL-4 levels [pg/ml] (mean ± SD)	3.78 ± 2.23	3.80 ± 2.30	3.66 ± 1.90
IL-5 levels [pg/ml] (mean ± SD)	3.14 ± 2.45	2.75 ± 1.98	4.99 ± 3.45
IL-10 levels [pg/ml] (mean ± SD)	6.54 ± 26.75	6.90 ± 29.31	4.83 ± 6.25
IL-12 levels [pg/ml] (mean ± SD)	14.35 ± 18.79	11.41 ± 17.01	28.26 ± 20.73
IL-13 levels [pg/ml] (mean ± SD)	6.20 ± 6.46	5.76 ± 6.46	8.26 ± 6.13
GM-CSF levels [pg/ml] (mean ± SD)	35.01 ± 20.08	33.73 ± 18.53	42.98 ± 25.01
Time of blood drawings (mean ± SD)	11:51 ± 2:28	11:56 ± 2:32	11:26 ± 2:10

### CRP, cytokines and depression symptoms

Linear regression analyses showed a significant increase in the severity of depressive symptoms associated with higher logTNF-α, logIFN-γ, logIL-5, logIL-12, and logIL13 (Table 3). The BDI-II sum score increased by a coefficient of up to 15.5 for each standard deviation of logIFN-γ, followed by 9.2 for each standard deviation of logTNF-α, by a coefficient of 9.2 for logIL-5, 6.7 for logIL-12 and 6.4 for logIL-13. Scores on the somatic-affective and cognitive dimensions of the BDI-II were significantly associated with logTNF-α, logIFN-γ, logIL-5, logIL-12, logIL13 and logGM-CSF. No significant association between the sum scores, the dimensions of depression and the cytokine levels were observed for logIL-2, logIL-4 or logIL-10 and also logCRP.

**Table 3:** Regression analyses between inflammatory agents and dimensions of depression.

	**BDI-II sum score**	**Somatic-affective dimension**	**Cognitive dimension**
_log_CRP	6.109 (−6.737 to 18.955) = 0.350	3.574 (−3.663 to 10.811) = 0.331	1.788 (−3.795 to 7.370) = 0.528
_log_TNF-α	**9.212 (3.017 to 15.408)** = 0.004^*^	**6.011 (2.528 to 9.494)**,<0.001^*^	**3.849 (1.116 to 6.582)** = 0.006^*^
_log_IFN-γ	**15.517 (10.463 to 20.571)**,<0.001^*^	**8.723 (5.854 to 11.593)**,<0.001^*^	**5.939 (3.697 to 8.180)**,<0.001^*^
_log_IL-2	1.974 (−1.107 to 5.056) = 0.208	1.160 (−0.576–2.897) = 0.189	0.956 (−0.383 to 2.294) = 0.161
_log_IL-4	3.219 (−4.954 to 11.392) = 0.438	2.216 (−2.425 to 6.858) = 0.348	1.801 (−1.753 to 5.354) = 0.319
_log_IL-5	**9.248 (4.664 to 13.832)**,<0.001^*^	**5.904 (3.341 to 8.468)**,<0.001^*^	**3.244 (1.228 to 5.260)** = 0.002^*^
_log_IL-10	3.437 (−0.445 to 7.318) = 0.082	1.998 (−0.193 to 4.190) = 0.074	1.387 (−0.300 to 3.074) = 0.107
_log_IL-12	**6.743 (3.647 to 9.839)**,<0.001^*^	**3.872 (2.118 to 5.626)**,<0.001^*^	**3.108 (1.749 to 4.466)**,<0.001^*^
_log_IL-13	**6.370 (1.900 to 10.840)** = 0.005^*^	**3.904 (1.388 to 6.420)** = 0.003^*^	**2.860 (0.897 to 4.823)** = 0.005^*^
_log_GM-CSF	**5.531 (0.119 to 10.944)** = **0.045**	**3.198 (0.150 to 6.246)** = **0.040**	**2.839 (0.491 to 5.187)** = 0.018^*^

*Coefficient (95%-CI), p-value. Significant correlations are highlighted in bold font. Significance after correction for multiple comparisons is labeled with an asterisk*.

### CRP, cytokines and neuroticism

Linear regression analyses between the pro-and anti-inflammatory markers, the six different dimensions of neuroticism and the sum score of neuroticism showed that a significant increase in the magnitude of neuroticism (NEO PI-R-N sum score) was dependent on higher logIFN-γ, logIL-5 and logIL-12 after correcting for multiple testing (Table 4). The increase in the anxiety dimension was predicted by logIFN-γ and logIL-12 levels. The depression dimension was found to significantly depend on logIFN-γ, logIL-5, and logIL-12. The self-consciousness dimension depended significantly on all parameters except logCRP and logIL4. The stress vulnerability dimension depended on logTNF-α, logIFN-γ, logIL-5, logIL-10, logIL-12, and logIL-13. The hostility dimension and the impulsiveness dimension were not associated with any of the cytokines.

**Table 4:** Regression analyses between inflammatory markers and the dimensions of neuroticism.

	**NEO-PIR-N sum score**	**Anxiety dimension**	**Hostility dimension**	**Depression dimension**	**Self-consciousness dimension**	**Impulsiveness dimension**	**Stress vulnerability dimension**
_log_CRP	8.937 (−22.468 to 40.341) = 0.575	5.052 (−1.738 to 11.843) = 0.144	1.826 (−4.392 to 8.044) = 0.563	−0.579 (−9.097 to 7.939) = 0.894	0.461 (−5.766 to 6.688) = 0.884	−0.580 (−5.205 to 4.044) = 0.805	2.757 (−4.130 to 9.643) = 0.431
_log_TNF-α	**16.571 (1.300 to 31.842)** = **0.034**	2.985 (−0.344 to 6.313) = 0.079	0.616 (−2.440 to 3.673) = 0.691	3.668 (−0.487 to 7.822) = 0.083	**4.797 (1.810 to 7.784)** = 0.002^*^	0.678 (−1.593 to 2.948) = 0.557	**3.828 (0.481 to 7.175)** = 0.025^*^
_log_IFN-γ	**31.712 (19.046 to 44.378)**,<0.001^*^	**6.363 (3.588 to 9.138)**,<0.001^*^	**3.474 (0.864 to 6.084)** = **0.009**	**8.134 (4.679 to 11.588)**,<0.001^*^	**5.510 (2.965 to 9.054)**,<0.001^*^	1.604 (−0.356 to 3.564) = 0.108	**6.628 (3.833 to 9.423)**,<0.001^*^
_log_IL-2	3.972 (−3.561 to 11.505) = 0.300	0.618 (−1.020 to 2.256) = 0.458	−0.574 (−2.072 to 0.24) = 0.451	0.910 (−1.149 to 2.969) = 0.384	**1.630 (0.140 to 3.119)** = 0.032^*^	0.800 (−0.314 to 1.914) = 0.158	0.724 (−0.945 to 2.392) = 0.394
_log_IL-4	6.204 (−13.759 to 26.167) = 0.541	1.632 (−2.702 to 5.966) = 0.459	−1.858 (−5.806 to 2.090) = 0.355	0.575 (−4.840 to 5.990) = 0.834	3.164 (−0.771 to 7.099) = 0.114	1.094 (−1.842 to 4.031) = 0.463	1.598 (−2.782 to 5.977) = 0.473
_log_IL-5	**17.439 (6.074 to 28.803)** = 0.003^*^	**2.823 (0.330 to 5.316)** = **0.027**	1.774 (−0.513 to 4.062) = 0.128	**4.223 (1.129 to 7.317)** = 0.008^*^	**3.833 (1.593 to 6.074)**,<0.001^*^	0.764 (−0.943 to 2.470) = 0.379	**4.021 (1.533 to 6.510)** = 0.002^*^
_log_IL-10	7.796 (−1.689 to 17.281) = 0.107	1.608 (−0.453 to 3.669) = 0.126	−0.171 (−2.061 to 1.719) = 0.859	1.710 (−0.867 to 4.286) = 0.192	2.159 (0.291 to 4.027), 0.024^*^	0.296 (−1.108 to 1.700) = 0.678	**2.194 (0.121 to 4.267)** = 0.038^*^
_log_IL-12	**14.806 (7.183 to 22.429)**,<0.001^*^	**2.530 (0.852 to 4.208)** = 0.003^*^	1.205 (−0.349 to 2.759) = 0.128	**4.074 (2.010 to 6.138)**,<0.001^*^	**3.059 (1.554 to 4.565)**,<0.001^*^	0.601 (−0.557 to 1.760) = 0.307	**3.336 (1.666 to 5.006)**,<0.001^*^
_log_IL-13	**13.038 (2.062 to 24.014)** = **0.020**	2.276 (−0.119 to 4.671) = 0.062	0.918 (−1.281 to 3.116) = 0.412	2.934 (−0.053 to 5.922) = 0.054	**3.329 (1.173 to 5.484)** = 0.003^*^	0.637 (−0.998 to 2.217) = 0.443	**2.945 (0.538 to 5.351)** = 0.017^*^
_log_GM-CSF	9.703 (−3.574 to 22.980) = 0.151	1.708 (−1.181 to 4.597) = 0.245	0.436 (−2.205 to 3.078) = 0.745	1.625 (−1.984 to 5.235) = 0.376	**3.148 (0.540 to 5.757)** = 0.018^*^	0.703 (−1.258 to 2.665) = 0.480	2.081 (−0.833 to 4.996) = 0.161

*Coefficient (95%-CI), p-value. Significant correlations are highlighted in bold font. Significance after correction for multiple comparisons is labeled with an asterisk*.

### Relationship between neuroticism, depressive symptoms and inflammatory markers

In a first step, the residuals of the BDI-II scores were predicted by the residuals of the NEO-scores. In a second step, the residuals of the cytokine levels (logTNF-α, logIFN-γ, logIL-5, logIL-12, logIL-13) were predicted by the residuals of the NEO-scores. In a third step, the residuals of the BDI-II-scores were predicted by the residuals of the cytokine levels and the NEO-scores. The results of the regression analyses are presented in Table 5

**Table 5:** Mediation analyses Regression analyses between dimensions of depression, neuroticism and the inflammatory agents.

**Criterion**	**Predictor**	**β**	***p***
BDI-II	NEO	1.60	< 0.001
TNF-α	NEO	16.42	0.029
IFN-γ	NEO	32.11	< 0.001
IL-5	NEO	18.38	0.002
IL-12	NEO	15.07	< 0.001
IL-13	NEO	4.61	0.016
BDI-II	TNF-α	0.009	0.048
	NEO	1.566	< 0.001
BDI-II	IFN-γ	0.017	0.005
	NEO	1.448	< 0.001
BDI-II	IL-5	0.011	0.016
	NEO	1.54	< 0.001
BDI-II	IL-12	0.011	0.026
	NEO	1.527	< 0.001
BDI-II	IL-13	0.0004	0.123
	NEO	1.572	< 0.001

*β, standardized coefficient of regression*.

**Figure 1:**
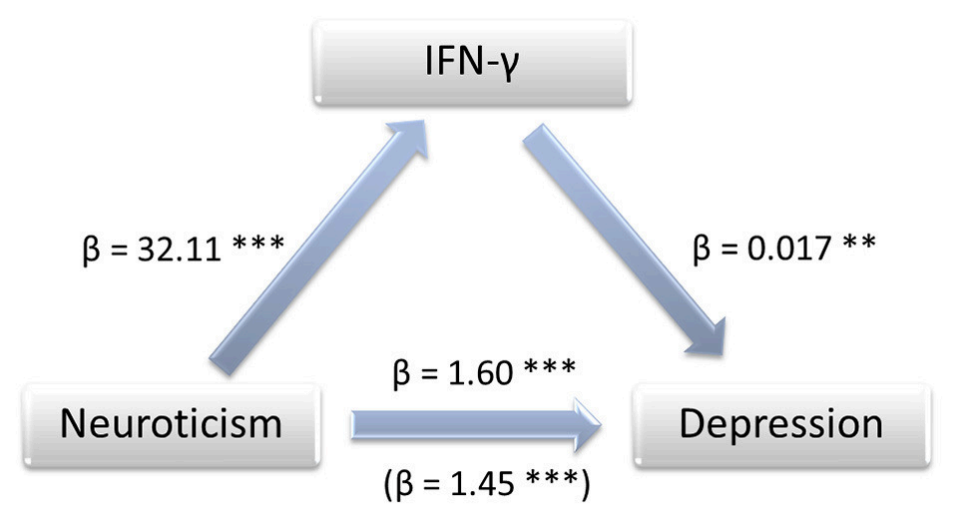
Mediation model showing the impact of IFN-γ on the association between neuroticism and depression. Annotations: β, standardized coefficient of regression, **p < 0.01, ***p < 0.001.

The bootstrapping analysis with *m* = 5,000 estimates revealed a significant indirect effect of the residuals of the NEO-scores on the residuals of the BDI-scores through the residuals of logIFN-γ (95% CI 0.0097, 0.0462), logIL-5 (95% CI 0.0027, 0.0230) and logIL-12 (95% CI 0.0013, 0.0291) -residuals. As an example, the standardized coefficients of regression for logIFN-γ are depicted in Figure 1. The Sobel-Z-test revealed a significant effect for the residuals through logIFN-γ (*Z* = 2.88, *p* = 0.004) that was trending for logIL-5 (*Z* = 1.89, *p* = 0.059) and IL-12 (*Z* = 1.91, *p* = 0.056).

## Discussion

The current findings on the relationship between both pro- and anti-inflammatory markers and characteristics of depression and neuroticism confirmed our hypothesis of a positive association between inflammatory agents and the degree of depression and neuroticism. The results further revealed that markers of inflammation may be significant mediators for the positive relationship between neuroticism and depressive symptoms.

After correcting for multiple testing and controlling for potential confounding variables, the analyses revealed that depressive symptoms were association with the cytokines TNF-α, IFN-γ, IL-5, IL-12, and IL-13. These results expand on the range of cytokines previously investigated. They also add to existing findings suggesting that a pro-inflammatory state is related to depressive symptoms in the general population as well as in cohorts of depressed subjects [e.g., (15–17, 31)]. Extending on previous research that focussed on pro-inflammatory markers, our analyses could not demonstrate an affection of the degree of depressive mood and cognition by the anti-inflammatory mediators IL-4 and IL-10. Though anti-inflammatory cytokines, have been found to be elevated in major depression and in response to pro-depressive agents (24, 32, 33), the degree of depression could previously not be statistically explained by IL-10 or IL-4 (24, 31). However, the sample sizes here and in previous studies may, in part, account for the negative results for which small effect sizes were to be expected (34). Results of a decreased IL-4-responsiveness of microglia as well as the inhibition of IL-10-signaling leading to depression-like behavior in animal models of depression demonstrate the need for further investigations on the role of anti-inflammatory agents in depression (35, 36).

The present results do not support the involvement of CRP as a relevant mediator once relevant covariates were accounted for (13, 15). In line with this, most of the mechanisms involved in the inflammation-depression -relationship have been described for TNF-α and IFN-γ, but not CRP, including the stimulation of the indolamine-2,3-dioxygenase (IDO) (10), the relationship with the psychopathology of depression (37–39) and the potential as an antidepressant drug target (14, 40). As for CRP, the differences in results between correlation and regression analyses (41) and the variability in results across other studies, showing no relationship between cytokines and depressive symptoms (20, 24), illustrates the need to account for potential confounders. Studies not including essential confounding parameters, such as BMI, smoking habits and inflammation-modulating drugs, should therefore be considered with caution.

Overall neuroticism and also several dimensions of neuroticism, in particular the self-consciousness and the stress vulnerability dimensions, were related to multiple cytokines, such as IFN-γ and TNF-α; however no such relationship was observed for the anti-inflammatory IL-4 and IL-10. These associations highlight the relationship between personality traits leading to long-term impairments in stress response and the regulation of mediators of inflammation. There is little data to which we can compare our findings; however, for CRP, the absence of associations are in line with a recent meta-analysis showing CRP and IL-6 not to be related to neuroticism (6). To our knowledge, this is the first study in the English language to examine the relationship between neuroticism and the anti-inflammatory cytokines IL-4 and IL-10, which warrants replication. For TNF-α, the findings from the limited number of previous studies conducted are consistent with ours; for example, in patients with hepatitis C, TNF-α correlated with scores of neuroticism after therapy (4). Hypothetically, the finding of up-regulation of markers of inflammation in neuroticism may explain the increased incidence of a wide range of physical illnesses in people vulnerable to neuroticism (42). Also, neuroticism may be associated with increased cytokine levels due to its relationship with a higher prevalence of obesity and smoking habits, factors known to up-regulate pro-inflammatory cytokines (9, 43, 44).

With regards to our findings of a relationship between neuroticism and depressive symptoms, neuroticism has repeatedly been shown to be associated with or to be a risk factor for the development of depressive disorders (7–9). To the best of our knowledge, this is the first time that inflammatory markers have been identified as a relevant mediator of this association. One possible patho-biological pathway for this link could be hypothalamic-pituitary-adrenal (HPA) axis activity: chronic stress induces the upregulation of HPA axis activity as well as the synthesis of cytokines (45–47). Some cytokines, for example IFN-γ, in turn stimulate the HPA axis (48). Of note, HPA axis activity has been found to be altered both in major depression and neuroticism (45). Other identified links between neuroticism and depression include a decreased expression of brain-derived neurotrophic factor (BDNF), as well as the activity of the anterior cingulate cortex [ACC; (45, 46)], for which an involvement of cytokines has been described (11, 49). Regarding the integration of this relationship into antidepressant treatment strategies, it should be of note that both inflammation (13, 18) and neuroticism (50, 51) impact on the treatment outcome of depression. Therefore, depressed patients with high neuroticism may require more specialized clinical efforts (50). In addition, the determination of inflammatory mediators as well as factors associated with increased cytokine levels and neuroticism, such as obesity and smoking should be taken into account (9, 19, 43, 44). Psychotherapeutic interventions may exert anti-depressant effects via modulation of neuroticism (52), however the question as to whether this is related to observed reductions of inflammation (53) is yet unknown.

This study needs to be considered in light of several limitations: The analyses were performed in participants with a high proportion of obesity which is unlikely to represent the general population. The sample size of the group of depressed patients was too small to perform separate analysis in this group and to rule out type II errors. This may also be the case for the total number of participants and potential effects with small effect sizes, like the relationship between depressiveness and serum levels of IL-4 and IL-10.

Our results show a relationship between depressive symptoms, neuroticism and cytokine levels, but cannot provide information on the molecular mechanisms underlying this association. A longitudinal design could more clearly demonstrate whether the personality trait neuroticism contributes to the risk of developing depression. Further, our analyses highlight the importance of including several potential covariates into the statistical analyses, such as BMI, medication status, and concurrent physical illnesses, due to their potential impact on inflammation. However, a more distinct impact, e.g., of the dosage of the separate drugs or the activity and acuity of the diagnoses, remains unclear.

In conclusion, the current study included 37 depressed and 175 non-depressed subjects, finding significant associations between depressive symptoms, the degree and dimensions of neuroticism and serum levels of pro-inflammatory, but not anti-inflammatory, cytokines. Further, IFN-γ, IL-5 and IL-12 were found to be significant mediators of the effect of neuroticism on depressive symptoms. These findings support the relationship between depressive psychopathology and pro-inflammatory conditions. Neuroticism as a long-term psychological stressor may exert pro-depressive features by facilitating a persistent low-grade inflammation. Since neuroticism and inflammation are related to the course of depression, psychotherapeutic emphasis on neuroticism and pharmacological targeting of inflammation may contribute to more personalized antidepressant therapeutic interventions, helping to prevent therapeutic non-response and the development of a chronic course of illness.

## Author contributions

FS and HH designed the study. FS, CS, and HH wrote the manuscript. LH and DT conducted the chemical analyses. FS and RM performed the statistical analyses. JM, RM, BD, and UH revised the manuscript. All authors approved the final manuscript.

### Conflict of interest statement

The authors declare that the research was conducted in the absence of any commercial or financial relationships that could be construed as a potential conflict of interest.
